# Communities of Primary Producers in the Series of Reservoirs on the Sava River (Slovenia)

**DOI:** 10.3390/plants14111665

**Published:** 2025-05-30

**Authors:** Igor Zelnik, Larisa Vodopivec, Mateja Germ

**Affiliations:** Department of Biology, Biotechnical Faculty, University of Ljubljana, Jamnikarjeva 101, 1000 Ljubljana, Slovenia; larisavodopivec96@gmail.com (L.V.); mateja.germ@bf.uni-lj.si (M.G.)

**Keywords:** diatoms, macrophytes, river reservoirs, phytoplankton, phytobenthos

## Abstract

Different communities of primary producers were surveyed in a series of five river reservoirs on the Sava River in southeast Slovenia. Seasonal differences of phytoplanktonic and phytobenthic communities were studied in the reservoirs of hydroelectric power plants. Macrophytes were surveyed in the summer, while phytoplankton and periphyton were sampled in the winter and summer of 2021. The taxonomic composition of diatoms was studied in greater detail and additionally analysed. The results showed that the species composition of phytoplankton and phytobenthos differed between seasons. The communities were also more similar between the seasons at the same sampling site than between the sampling sites. Temperature was the parameter that had the greatest impact on the taxonomic composition of phytoplanktonic and phytobenthic communities. In total, 51 algal taxa and 81 species of diatoms were recorded, respectively. Furthermore, 15 species of aquatic macrophytes were found. The abundance of phytoplankton was the highest in the lowest reservoir in the series, in Brežice, where the temperature was also the highest. The number of macrophytes also increased downstream, but their abundances were low, mainly due to coarse substrate on the banks consisting of rocks and boulders. Constructing additional reservoirs on this section of the Sava River could increase the probability of the substantial growth of phytoplankton and cyanobacteria within it. On the other hand, providing patches of finer substrates (gravel, pebbles) would support the abundance of macrophyte species, which could control the concentrations of nutrients in the summer and prevent the blooms more efficiently.

## 1. Introduction

Rivers are important aquatic ecosystems because of their dynamic flow and diverse habitats, hosting a very high level of biodiversity. River flow enables the transport of organic and inorganic matter and pollutants on a local and regional scale. The river’s hydromorphological properties greatly impact the distribution of dissolved gases, nutrients, and organisms [1]. Aquatic organisms living in river ecosystems must be adapted to water currents, movements of the sediments, and burial [2]. Dams on rivers have significantly altered their dynamics, including river connectivity, hydrologic and thermal regimes, sediment and soluble substances, and channel morphology. These changes adversely affect the quality and quantity of aquatic life habitat [3]. Cascading hydropower dams affect riverine ecology even more severely than a single dam [4], as they create a series of reservoirs, increasing the retention time of water, and its more intense exposure to solar radiation influencing the conditions. Such artificial reservoirs are very complex systems and are important from an ecological, economic, and social point of view. However, reservoirs have become a new habitat for numerous taxa of primary producers, inhabiting the littoral zone as phytobenthos and macrophytes, as well as the water column as phytoplankton.

Algae, as primary producers, play an important role in aquatic ecosystems [5], including reservoirs. Phytoplankton represents a base for the food webs in water bodies [6], where the retention time of the water is sufficient to enable its development. In reservoirs, the water retention time is usually long enough for phytoplankton to develop. Therefore, the development of the phytoplankton merits more attention in these ecosystems. Phytoplankton communities can be used as indicators to assess aquatic habitat transition and ecosystem degradation [7]. The operation of dams severely affects phytoplankton communities and their abundance [8]. Cyanobacteria often form blooms in nutrient-rich reservoirs that, in certain conditions, can be the source of cyanotoxins, which are bioaccumulated in aquatic organisms [9]. Cyanotoxins are harmful to aquatic biota but can also threaten humans, either directly or via water supply networks. Diatoms are important mediators of human impacts, since they are usually the most abundant taxonomic group in the phytobenthos. They are builders and stabilisers of the biofilm, as many species are firmly attached to the substrate and have an anchoring function. The epilithic diatom communities are more stable than others due to the stability of their substrate [10]. Diatoms are adapted to poor light conditions and turbulent water flow due to the synthesis of silica cell walls [11]. Diatoms in phytoplankton of the reservoirs are often drifted by the current from the upstream sections, but if the conditions are stable enough, the population of planktonic species also develop.

Cibils Martina et al. [12] studied the effect of a dam on epilithic algal communities. They discovered that floods caused the sloughing from the outer layers of the biofilm, placing the algal community in an early successional stage [12]. The results showed that the impacts of dam construction on benthic algal communities are seen after 2 to 3 years [13]. Aquatic macrophytes play crucial roles in river ecosystems, influencing flow, water chemistry, and sediment dynamics [14]. They also offer shelter for many other organisms [15] and act as a food source [16]. Macrophytes are important for the conservation of aquatic ecosystems and affect the diversity of other biotic communities [17]. Slow-flowing habitats upstream of dams might trap drifting propagules of macrophytes and interrupt hydrochory and ichthyochory, since upstream dispersal is crucial to maintaining macrophyte abundance and distribution [14].

The aim of this study was to determine the differences in algal communities in a series of reservoirs on the Sava River (southeast Slovenia). We hypothesised that there would be more significant differences in phytoplankton and phytobenthic algal communities between the seasons than between reservoirs. One of the reasons for this is the difference in water temperature and the amount of nutrients. We were also interested in how the series of reservoirs in the Sava River affects the development of potentially toxic cyanobacteria.

## 2. Results

### 2.1. Phytoplankton Community Composition

**Figure 1:**
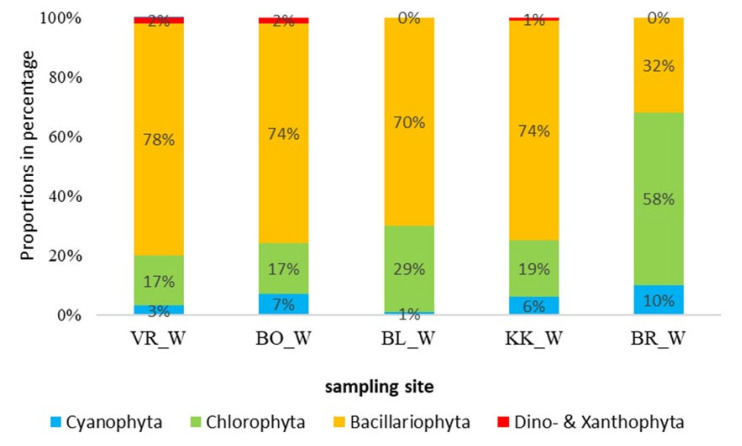
Phytoplankton community composition in the reservoirs in the lower Sava in March. Abbreviations: VR—HPP Vrhovo reservoir; BO—HPP Boštanj; BL—HPP Blanca; KK—HPP Krško; BR—HPP Brežice reservoir; _W—winter samples.

The proportions of higher taxa in phytoplankton in March are seen in Figure 1. In all the samples, with the exception of BR (the lowest HPP of the series), we found the highest proportion of diatoms, between 70 and 78%. Among the diatoms, the genus *Navicula* was represented with the highest proportion. Diatoms were followed by green algae, which represented between 17% and 29% at all sample sites. Green algae dominated in BR, reaching up to 58%. The genera *Microspora* and *Coelastrum* were observed in all samples. All sampling sites contained less than 10% cyanobacteria. Of all the identified species or genera in the samples, more than half of the taxa belonged to diatoms.

**Figure 2:**
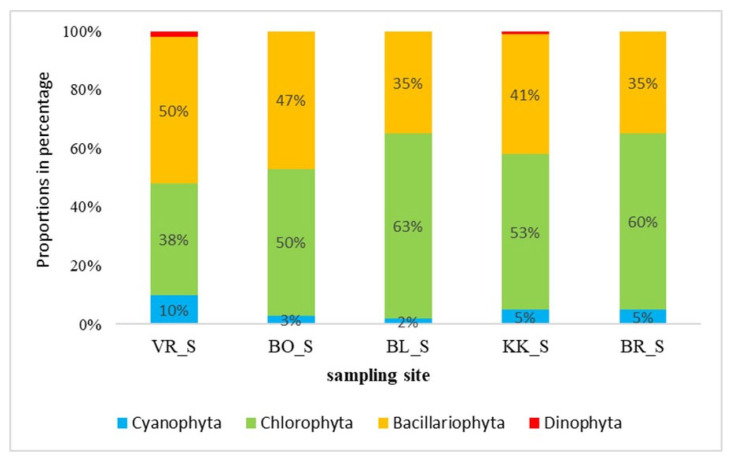
Phytoplankton community composition in the lower Sava in July (_S—summer samples). Abbreviations are explained in the caption to Figure 1.

**Figure 3:**
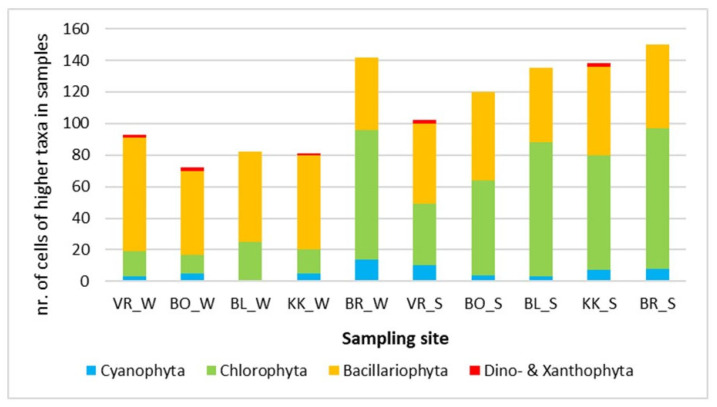
Abundance of phytoplankton in the reservoirs in March—winter samples (_W) and in July—summer samples (_S). Other abbreviations: VR—HPP Vrhovo reservoir; BO—HPP Boštanj; BL—HPP Blanca; KK—HPP Krško; BR—HPP Brežice reservoir.

Figure 2 shows the composition of phytoplankton algae in July. In summer, green algae dominated in most samples, which were represented in the range of 50% to 63%, except VR (the first of the studied reservoirs in the series), where they made up 38% of all algae in the sample. Their increase downstream is evident in Figure 3, where abundances are displayed. Among the green algae, there was a great variety of taxa compared to the winter sample (Table 1, Table S1). On VR, as much as 50% of algae in phytoplankton belonged to diatoms. At the other sampling sites, diatoms represented between 35 and 47%. Cyanobacteria had much lower proportions than green algae and diatoms. Again, they represented less than 10% of the sample. The most common genus found in all the samples was *Phormidium*.

Table 1 shows the proportions of taxa of the phytoplankton community that dominated the samples. In most samples from March, diatoms of the genera *Navicula* and *Nitzschia* dominated. The species *Diatoma vulgaris* dominated in VR and in KK (Table S2). BR stands out the most, where the representative of the green algae *Microspora* dominated. In July, the genus *Cyclotella* prevailed among diatoms, which was dominant in all sample sites except for BL. In July, representatives of green algae dominated, namely, the genera *Coelastrum* and *Scenedesmus* and the species *Pediastrum duplex* and *Scenedesmus quadricauda* (Table 1).

### 2.2. Phytobenthos Community Composition

**Figure 4:**
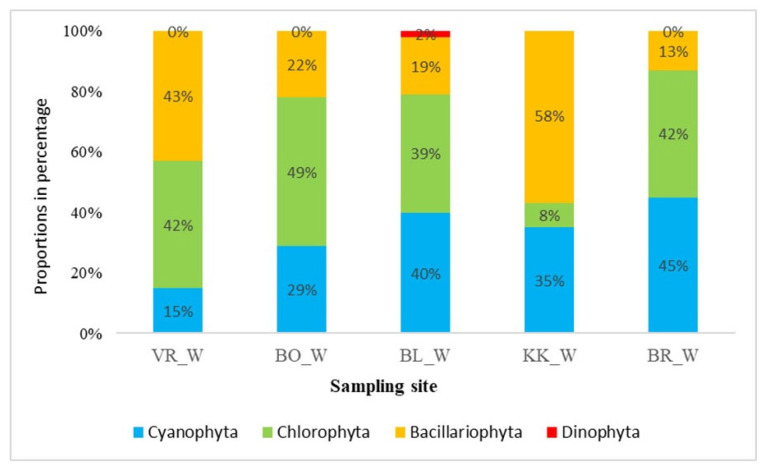
Composition of phytobenthic algae in March. Abbreviations: VR—HPP Vrhovo reservoir; BO—HPP Boštanj; BL—HPP Blanca; KK—HPP Krško; BR—HPP Brežice reservoir; _W—winter samples.

Figure 4 shows the composition of the phytobenthic community in March. The composition of the periphyton is very similar at all sample sites, except in KK. Green algae dominated and presented 42–49%, with the exception of KK, where green algae presented only 8%. Diatoms dominated in KK, representing as much as 58%. In other sites, their share was smaller, namely 13–43%. Cyanobacteria were represented in the phytobenthos with a larger proportion than in phytoplankton, and their proportion increased downstream from reservoir VR to the lowest HPP BR, where the proportion of cyanobacteria was 45%. More than half of the taxa in the phytobenthic community belonged to diatoms.

**Figure 5:**
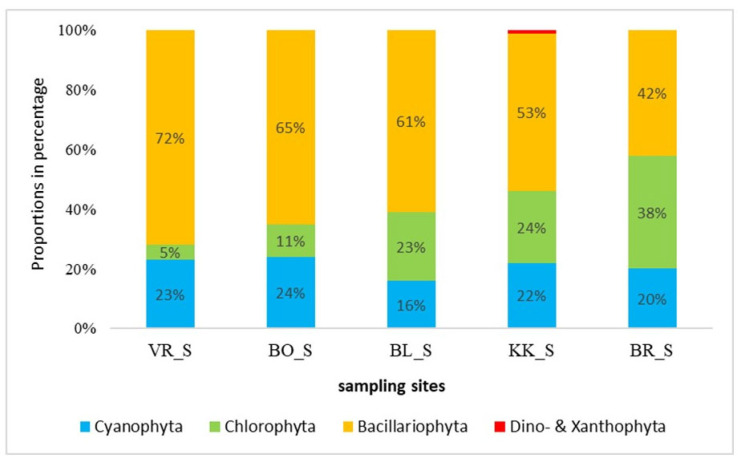
Composition of the phytobenthic community in July (_S—summer samples). Abbreviations are explained in the caption to Figure 4.

Figure 5 shows the composition of the phytobenthic community in July. Diatoms had the highest proportion in the phytobenthic community in all samples, and their abundance decreased from VR (the first of the studied HPP in the series) to BR (the last of the studied HPP in the series). They were most frequently represented in VR, where their share was 72%, and the smallest in BR with 42%. Cyanobacteria accounted for around 20% at all sampling sites. Cyanobacteria were more diverse in the summer than the winter samples. The proportion of green algae increased from VR (5%) to BR (38%).

In March, the cyanobacteria of the genus *Oscillatoria* and green alga of the genus *Microspora* were dominant in phytobenthos at all sampling sites, except in KK (Table 2 and Table S3). Of the diatoms, the genus *Navicula* dominated in KK, and the genus *Nitzschia* dominated in VR. In July samples, the cyanobacteria genus *Oscillatoria* also dominated at four sites. Among diatoms, the species *Diatoma ehrenbergii* and representatives of the genera *Navicula* and *Nitzschia* dominated at VR and BL.

Table 3 shows which physical and chemical parameters influence the species composition of phytoplankton and phytobenthic communities and which differ statistically significantly between the summer and winter sampling. We can notice the gradients in some of the parameters; for instance, the concentration of nitrates and orthophosphates decreases downstream in the winter samples, whereas pH increases downstream, particularly in summer.

### 2.3. Comparisons of the Algal Communities

**Figure 6:**
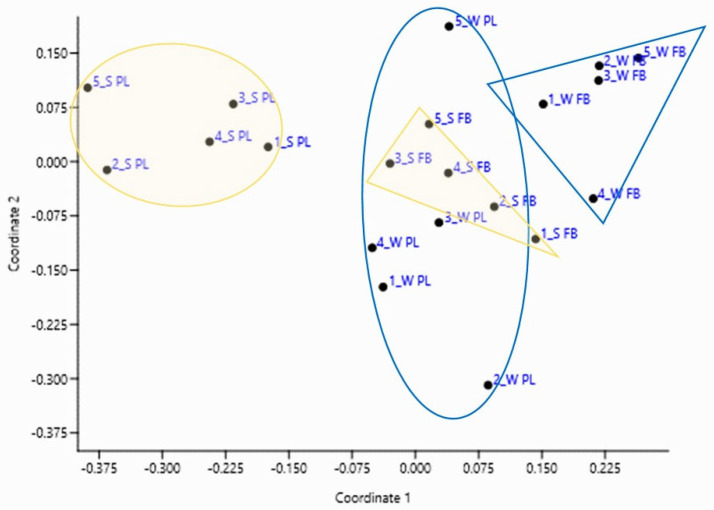
Ordination of the phytoplanktonic (PL) and phytobenthic (FB) communities obtained with NMDS and Bray–-Curtis similarity index (the stress level: 0.1882). W—winter, S—summer, 1—HPP Vrhovo reservoir, 2—HPP Boštanj, 3—HPP Blanca, 4—HPP Krško, 5—HPP Brežice. Blue ellipse represents the winter phytoplanktonic community, and blue triangle represents the phytobenthic. The yellow ellipse represents the summer phytoplanktonic community, and the yellow triangle represents the phytobenthic one.

We compared the composition of the phytoplanktonic and phytobenthic communities. The composition of the studied communities was relatively similar, but the most specific and distinctive, respectively, was the composition of the planktonic community in summer (Figure 6). The phytoplanktonic community sampled in July was characterised by eu-planktonic taxa such as *Microcystis* sp., *Pediastrum duplex* and *Cyclotella* sp., occurring with considerably higher constancy and abundance in the mentioned samples. On the contrary, the phytobenthic community sampled in March was characterised by a high abundance of the diatoms *Navicula lanceolata* and *Nitzschia brevissima* (Table S4). As presented in Figure 6, the planktonic community in March was more similar to phytobenthic communities due to less favourable conditions for the growth of planktonic species. We noticed the differences in average discharge and water temperature in a three-week period before the sampling, which is most crucial for the development of planktonic and benthic algal communities [18]. The average discharge before the sampling in March was 25% higher than in July, whereas the water temperature was 9.3 °C in March, compared to 23 °C in July. We should also consider the light intensity, which is stronger on 6 July, a 15-day shift from the summer solstice, compared to the light on March 18, which is three days before the equinox. These factors create more favourable conditions for the development of phytoplankton, especially when the green algae are concerned.

### 2.4. Influence of Environmental Factors on the Composition of the Phytoplanktonic and Phytobenthic Communities

Water temperature was the factor that statistically significantly explained the taxonomic composition of all four data sets (see Table 4)—in phytoplanktonic and phytobenthic communities, as well as in diatom metacommunities, which were analysed additionally. Besides the water temperature, a significant influence on the composition of specific metacommunities (see Table 4) was confirmed for pH, the concentration of nitrates and orthophosphate, and the order of the reservoir in the lower part of the river Sava reservoir series. Temperature explained between 26% and as much as 65% of the variability in the species composition of the specific communities (Table 4). These percentages were lower in the case of other factors.

### 2.5. Macrophyte Diversity

A total of 15 species of macrophytes were recorded at all sampling sites. A higher number of species was recorded at the lower sampling sites at HPP KK (10) and HPP BR (7). The lowest number of species was recorded at the sample sites BL and BO (Table 5), where only 4 species of macrophytes were recorded. *Myriophyllum spicatum* was present at all sampling sites, while an invasive alien species, *Elodea nuttallii*, was recorded in the lower three reservoirs (Table 5). We recorded three growth forms of macrophytes—natant, emergent and submerged macrophytes.

## 3. Discussion

Water retention time is substantially higher in reservoirs than in a free-flowing river, which affects the development and biomass of cyanobacteria and algae [19]. With additional reservoir(s) on the river, the cumulative retention time and temperature of the water increases, facilitating the growth of plankton and cyanobacterial populations. Cyanobacterial blooms in eutrophic ecosystems have many negative effects, including increased methane (CH_4_) emissions [20]. Cyanotoxins released from cyanobacteria threaten other organisms, including humans [21]. The temperature of the water in reservoirs is higher than in free-flowing rivers due to more intense exposure to solar radiation resulting from the longer retention time of water in a substantially wider and more exposed water surface in reservoirs compared to relatively more shaded free-flowing rivers. In our study, the temperature was the highest in the lowest reservoir of the River Sava, so we expected the highest abundance of phytoplankton and the number of macrophytes there, which was indeed the case. The reason for the increasing abundance of phytoplankton downstream is also the additive effect of the total retention time in a series of reservoirs, allowing the algae to reach greater population densities and their drift towards lower reservoirs.

### 3.1. Phytoplankton

The species composition of algae differed between reservoirs and seasons. Stevenson [22] reports that phytoplankton is usually dominated by diatoms during spring, while green algae and cyanobacteria may dominate during summer, which is in line with our results from the March samples. In the March samples, diatoms dominated at four sites, representing more than 70%, except for BR (the last studied HPP in the series). Diatoms dominate in winter mainly because they need less light for their growth than green algae. Diatoms are competitive at various nutrient concentrations, lower temperatures, and low light intensity [23]. Green algae accounted for cca 20%, except for BR, where their proportion was 58%. A smaller proportion belonged to Cyanobacteria, Xanthophyta, and Dinophyta. The opposite was the case in the summer, when all sites except VR (the first studied HPP in the series) were dominated by green algae, with proportions between 50 and 60%. Such high values can be attributed to higher summer temperatures and light intensity, accelerating the growth of green algae within the phytoplankton. A taxon, most characteristic of the summer phytoplankton, is *Scenedesmus quadricauda*, which has the highest percentage in the summer samples (13.9%) but is absent in winter. This taxon of green algae is also absent in phytobenthos and contributes most to the separation of the summer phytoplankton samples from the other three groups (see Figure 6). The conditions enabling the development of phytoplanktonic community in the summer are also evidenced by a high proportion of the eu-planktonic taxon *Cyclotella*, which was the second most common taxon in the summer samples, with 13.5% on average, but only 2.6% in winter, while it was rare in phytobenthic communities (<1%). In winter, the benthic genus *Navicula* dominated among the diatoms.

### 3.2. Phytobenthos

Simić et al. [24] studied the phytobenthos community along the Sava River in the summer and found that diatoms were the most abundant group across all samples. We can also confirm this since, unlike the phytoplankton, when green algae dominated in the summertime, the phytobenthic community was dominated by diatoms (42–72%). The proportion of diatoms was the highest at VR (the first studied HPP in the series) and decreased downstream towards BR (the last in the series) (Figure 4 and Figure 5). Many diatoms are carried with the flow of the Sava River, and the first reservoir is most impacted by that effect, which might explain this. The greater abundance of diatoms can be explained by water turbidity, which was higher in summer and in the upper reservoirs, reducing the light intensity and influencing the primary production of the phytobenthos [25]. Low light intensities favour the diatoms, which need less light for successful growth than other algae. The opposite situation was observed with green algae, since their proportion increased downstream. Cyanobacteria were represented with a larger proportion in the phytobenthos than in phytoplankton, and their proportion increased downstream in the March samples. Dalu and Wasserman [26] found that Cyanobacteria relative abundances were the greatest during the cold season.

*Diatoma ehrenbergii* was very abundant in phytobenthos in the first and third reservoir (VR, BL) in the summer (Table 2). The genera *Navicula* and *Nitzschia* were also common. Diatoms from the genera *Navicula* and *Nitzschia* are common in sites where fine particles are settling due to their high motility, enabling them to escape the effect of burial on the bottom. Diatoms of the genus *Navicula* are often found in turbid water [27], as was found in these reservoirs in summer. In March, we found the following dominant species among diatoms: *Achnanthidium minutissimum, Navicula lanceolata*, and *Nitzschia brevissima*. In both summer and winter, the species *Coccconeis placentula*, which is typical for eutrophic water bodies [28], was abundant. *Navicula lanceolata* and *Cyclotella meneghiniana* were dominant in KK in winter and in summer, as already reported by [29], who found that it dominated the phytobenthos in the samples collected below the dam of HPP Krško (KK).

Values of the saprobic index in the studied reservoirs were higher in the March samples than in the July ones. Zelnik & Sušin [29] similarly reported a higher saprobic index in winter than in summer for free-flowing sections of the Sava River. In both winter and summer, the species *Nitzschia palea*, which can tolerate high contents of DOM in water [30], was abundant in the samples.

The values of the trophic index classify all samples in the polytrophic aquatic environment, characterised by a high nutrient load [31]. *Navicula veneta* is found in environments with a high concentration of nutrients and is typical of aquatic ecosystems that are strongly influenced by human activity [32]. It was most common in the first reservoir VR, both in July and March, where the value of the trophic index was also the highest, indicating the influence of river inflow.

The similarity of communities is higher within the sampling season than according to the sampling site. Changes between the seasons were more evident than changes between sampling sites, as recorded in the River Savinja [33].

### 3.3. The Influence of Environmental Factors on the Composition of Algal Communities

The composition of phytoplanktonic and phytobenthic communities was statistically significantly influenced mainly by water temperature, pH, order of the reservoir downstream, and nitrate and orthophosphate concentration. The division of the algal communities into four groups is based on their occurrence in the water column or on the substrate from which we sampled all algae, as well as the exclusion of diatom metacommunities from both mentioned samples with a separate procedure. The importance of diatoms as bioindicators for the assessment of water quality is well known [34]. Their diversity and different responses to environmental factors were the reasons that many river quality indices are based on diatom data only [35,36]. In Slovenia, the phytobenthic diatom community is used for the determination of trophic index (TI) and saprobic index (SI), according to the Water Framework Directive (2000/60/EU), and enables the detection of nutrient and organic pollution, respectively [35].

Zepernick [37] evidenced that high pH negatively affected growth rate and diatom silica deposition and, thus, represented a competitive disadvantage for diatoms in lakes. In our study, the proportions of diatoms in phytoplankton decreased downstream (Figure 2 and Figure 3). In contrast, the abundance of the total phytoplankton increased (Figure 3) as did the pH of the water, particularly in July (see Table 3). In all four analyses of phytoplankton, phytoplankton diatoms, phytobenthos, and phytobenthic diatoms, water temperature affected the composition of algal communities (Table 4). In both samplings, there was a temperature gradient between the reservoirs within the same season. Temperature is one of the main physicochemical parameters affecting the distribution and ecology of periphyton [38]. Water temperature directly affects algal growth, as well as light and the availability of nutrients [39] by affecting the cellular composition, uptake of nutrients, photosynthesis, and growth [40,41]. Moreover, it was reported that increased temperature leads to the success of more harmful Cyanobacterial species, which form harmful algal blooms that have increased worldwide [42].

### 3.4. Macrophytes

We recorded 15 species of macrophytes at 5 sampling sites. The most common form was emergent macrophytes, while the submerged macrophytes were less represented in the reservoirs due to turbid water. Species diversity increased downstream, most likely due to higher water temperatures [43]. *Myriophyllum spicatum* was the only species present in all of the reservoirs. It has a wide geographical distribution [44] and is one of the most widespread species in Slovenian rivers [45].

Results from this study and the previous one showed competition between the alien species *Elodea nuttallii* and the autochthonous *M. spicatum*. *E. nuttallii* was found in the lower three reservoirs. On the contrary, the autochthonous species *M. spicatum* was present in all the reservoirs, with a slightly higher abundance in the upper three reservoirs (VR, BO, and BL) than in the lower two. We found that the abundance of *M. spicatum* negatively correlated with the abundance of the alien invasive species *Elodea nuttallii*. In the lowest reservoir BR, we also found a spatial separation of the two species. *E. nuttallii* thrived closer to the bank, while the *M. spicatum* grew deeper in the water. Mazej Grudnik & Germ [46] published similar findings; namely, in the HPP reservoirs on the Drava River, *E. nuttallii* was competitively more successful in parts that are less exposed to the main water flow, while *M. spicatum* prevailed in locations exposed to higher flow velocity. *E. nuttallii* is an invasive alien species in Slovenian watercourses [47] and has allelopathic potential, as it secretes substances into the water that limit the growth of other plant species [48]. We presume this species will probably continue to spread along the Sava River. Troia et al. [49] found that in mixed aquatic communities, plant density is a relevant factor affecting invasiveness, for instance, through competition. We think that the reservoirs with a low density of native species are endangered by the invasions of alien plants.

A relatively low abundance of macrophytes is a consequence of the fluctuation of the water level occurring in reservoirs, as evidenced by Zhao et al. [50], who found that the biomass and the coverage of submerged macrophytes decreased with water-level fluctuation. A coarse substrate in the littoral of the studied reservoirs, which was most extreme in the lowest reservoir BR, also negatively influences the thriving of macrophytes. Predominant boulders in the littoral of reservoirs are not a suitable substrate for the roots of vascular plants and store low amounts of nutrients.

At all sample sites, except for VR, we observed the presence of filamentous algae. Filamentous algae develop in late summer [51] and are common in eutrophic rivers [52] that have long residence times [53], which is characteristic of the studied reservoirs. Troia et al. [49] report that in aquatic environments, an important factor determining invasiveness is nutrient availability, and invasion would increase with eutrophication.

## 4. Materials and Methods

### 4.1. Study Area

**Figure 7:**
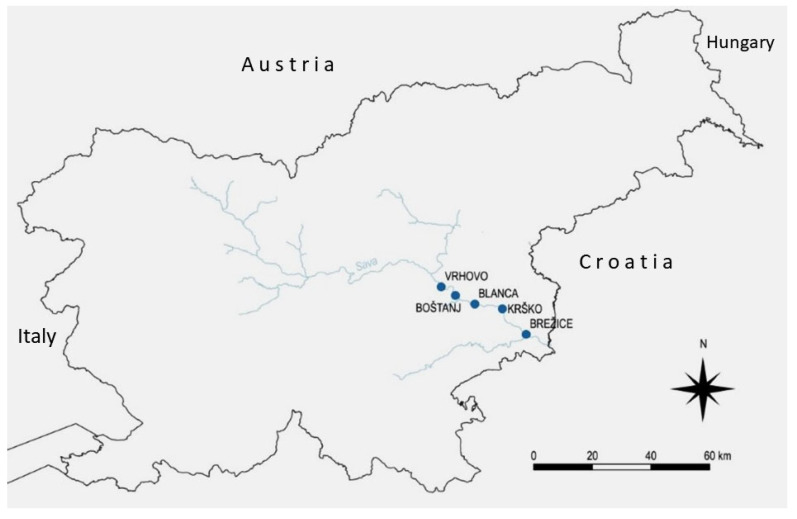
Map of sampling sites on the Sava River in Slovenia; the series of hydroelectric power plants on the lower Sava River HPP Vrhovo, HPP Boštanj, HPP Blanca, HPP Krško and HPP Brežice contributed to the creation of five reservoirs.

The Sava River is 945 km long and flows through the territories of Slovenia, Croatia, Bosnia and Herzegovina, and Serbia. It is the tributary of the Danube River with the largest discharge and has a significant ecological impact on the Danube basin [54]. A part of the Sava River in Slovenia, downstream of the confluence with the Savinja River, is called the lower Sava. The course of the lower Sava has been regulated with dams of five hydroelectric power plants, namely, HPP Vrhovo (VR), HPP Boštanj (BO), HPP Blanca (BL), HPP Krško (KKK), and HPP Brežice (BR), which turned the river into a series of reservoirs (Figure 7 and Table 6).

In each reservoir, we chose a sampling site 100–300 m upstream of the dams (Figure S1, the coordinates are listed in Table 6). Phytoplankton and periphyton samples were collected in March, representing winter samples, and in July, representing summer samples. Macrophytes were surveyed in summer only, since most of them are not developed in winter. On the same sampling sites, concurrently, we also measured physical and chemical parameters of the water. Temperature, pH, electrical conductivity, saturation with oxygen (%), and the concentration of dissolved oxygen in the water (mg/L) were measured using a multimeter PCD 650 (Eutech Instruments, Singapore). For the measurements of nitrates and orthophosphates, we collected water samples and stored them in a freezer until analysis. The first sampling of phytoplankton and periphyton and all mentioned parameters occurred on March 18, referred to as winter sampling, and the second on 6 July 2021, referred to as summer sampling.

### 4.2. Sampling Methods

The phytoplankton community was sampled with a plankton net (20 microns mesh size) by filtering 30 L of water from a depth of 0–30 cm, sampled approximately 1 m offshore, at each sample site. The sample was fixed with Lugol (standard solution).

Periphyton was sampled quantitatively. We selected five stones (6–20 cm in size) at each sampling site. Using a scalpel and a toothbrush, we scraped the periphyton from a rectangle measuring 2 cm × 3 cm from the stones. The samples were fixed with 37% formaldehyde in a ratio of 1:7.

On a 100-m section, we surveyed all types of macrophytes and determined their abundance. Abundance was assessed on a five-point scale from 1 to 5, where 1 means very rare, 2 rare, 3 moderately present, 4 common, and 5 very common, dominant species [55]. Macrophytes at greater depths were sampled using a telescopic rod with hooks, enabling their collection from the bottom.

The concentrations of nutrients were measured with HACH Lange tests. The cuvette HACH Lange tests were performed for the content of ammonium ions (LCK 304), nitrate (LCK 339), and orthophosphate (LCK 549). Concentrations were measured with a spectrophotometer HACH Lange DR 3900 (Loveland, CO, USA).

### 4.3. Preparation of Diatom Samples

Samples of the phytoplankton were homogenised with a magnetic stirrer, and a 5 mL subsample was boiled with 65% nitric acid to remove organic matter. Permanent slides were prepared by mounting cleaned frustules with Naphrax^®^ (Brunel Microscopes, Chippenham, Wiltshire, UK). Diatoms were identified at 1000× magnification using a light microscope (Olympus CX41 microscope, Tokyo, Japan). Due to the scarcity of diatoms in some samples, 200 frustules were identified to the species level and counted in each sample. The same procedure was used for the preparation of the permanent slides of the phytobenthos. In this case, a 2 mL subsample was boiled due to the much higher density of algae in the suspension. Diatom species were identified with keys published by [56,57,58,59,60]. The nomenclature of diatom taxa follows [56], with the exception of species from the genera *Cyclotella, Stephanodiscus* and *Aulacoseira*, where we followed [57,58,59,60].

### 4.4. Data Analyses

We calculated the trophic index (TI) and saprobic index (SI) based on the diatoms, according to [61,62], respectively. Multivariate analyses were performed with the CANOCO 4.5 software package [63]. Firstly, we analysed the composition of the phytoplankton and phytobenthic communities using detrended correspondence analysis (DCA) to see whether the taxon abundance data along the gradients are distributed linearly or unimodally. If the mentioned data were distributed linearly (eigenvalue of the 1st axis < 0.4), we chose redundancy analysis (RDA) below, but if they were distributed unimodally, then canonical correspondence analysis (CCA) was used. Either with RDA or CCA, we calculated which environmental factors we measured or evaluated had a statistically significant influence on the taxonomic composition of phytoplankton, phytobenthos, and diatom metacommunities. We used 10 variables for the analysis: order of the reservoir downstream, temperature, pH, conductivity, saturation of the water with oxygen, dissolved oxygen concentration, nitrate concentration, orthophosphate concentration, and SI and TI values. We used forward selection of these variables, with 499 permutations performed under the full model, to rank the relative importance of the variables and to avoid co-linearity. Only significant variables (*p* < 0.05) were considered in further analyses. We obtained information on which factors have a statistically significant influence on the distribution of algal taxa and the proportion of the variance of taxonomic composition they explain.

The similarity of taxonomic composition between the samples of the phytoplanktonic (PL) and phytobenthic (FB) communities was analysed with NMDS and Bray–Curtis similarity index and visualised with an ordination diagram in PAST [64].

## 5. Conclusions

Seasonal dynamics significantly influence the algal community in the plankton and periphyton in reservoirs on the Sava River in SE Slovenia. The compositions of the phytoplanktonic and phytobenthic algal communities differed between the seasons. Communities were more similar in the same season than between specific reservoirs. The compositions of the studied algal communities (phytoplankton, phytobenthos, and diatoms alone) were most strongly influenced by water temperature. A larger abundance of phytoplankton was present in the lower reservoirs, where the water temperature was also higher. Each additional reservoir, beside the existing series, would favour the growth of phytoplanktonic green algae and cyanobacteria, affecting other organisms. A higher abundance of macrophytes would positively influence the structure and function of these reservoirs, as they could absorb a significant amount of nutrients during the summer, which is the most critical period for the cyanobacterial blooms to occur. We noticed that the total abundance of macrophytes positively correlated with the conductivity of the water. Moreover, the abundance of the most common species, *Myriophyllum spicatum*, negatively correlated with the abundance of the alien invasive species *Elodea nuttallii*, which is another reason to facilitate the spread of native macrophytes in these reservoirs. However, the prerequisite is a more favourable substrate in the littoral zone of these reservoirs.

## Figures and Tables

**Table 1:** Proportions (in %) of dominant taxa of the phytoplankton community of all sites in winter samples (W) and summer samples (S). Abbreviations: VR—HPP Vrhovo reservoir; BO—HPP Boštanj; BL—HPP Blanca; KK—HPP Krško; BR—HPP Brežice. Highlighted in blue: dominant taxa in winter samples; Highlighted in red: dominant taxa in summer samples.

Sampling Site		VR		BO		BL		KK		BR	
Taxon/Sampling Season		W	S	W	S	W	S	W	S	W	S
**Cyanophyta**											
*Phormidium* sp.		1.1	8.8	0	2.5	0	2.2	0	2.9	9.9	2.0
**Chlorophyta**											
*Coelastrum* sp.		1.1	8.8	2.8	3.3	1.2	26.7	9.9	10.1	7.0	2.0
*Microspora* sp.		16.1	12.8	11.1	0	28.1	11.1	8.6	10.1	45.1	2.6
*Pediastrum duplex*		0	0	0	15.8	0	0	0	11.6	0	21.1
*Scenedesmus* sp.		0	3.9	0	3.3	0	11.9	0	11.6	0	21.1
*Scenedesmus quadricauda*		0	11.8	0	26.7	0	11.9	0	8.7	0	10.5
**Bacillariophyta**											
*Cyclotella* sp.		2.2	16.7	0	13.3	4.9	8.2	3.7	13.0	2.1	16.5
*Diatoma vulgaris*		15.1	9.80	0	15.8	4.9	7.4	16.1	5.8	4.9	2.0
*Navicula* sp.		31.2	7.8	9.7	1.7	18.3	0.7	18.5	0.7	7.8	0.7
*Nitzschia* sp.		7.5	3.9	23.6	3.3	19.5	3.7	7.4	2.9	4.2	3.3
*Surirella* sp.		5.4	3.9	29.2	0	1.2	0	0	1.45	0.7	0.7

**Table 2:** The proportions (in %) of dominant taxa of the phytobenthic community of all sites in winter samples (W) and summer samples (S). Abbreviations: VR—HPP Vrhovo reservoir; BO—HPP Boštanj; BL—HPP Arto-Blanca; KK—HPP Krško; BR—HPP Brežice. Highlighted in blue: dominant taxa in winter samples; Highlighted in red: dominant taxa in summer samples.

		VR		BO		BL		KK		BR	
Taxon/Season		W	S	W	S	W	S	W	S	W	S
**Cyanophyta**											
*Lyngbya* sp.		2.9	0	7.0	4.5	3.9	1.0	5.8	5.5	14.2	5.6
*Oscillatoria* sp.		12.2	20.9	21.9	17	33.9	9.3	29.2	13.2	30.6	14.8
**Chlorophyta**											
*Microspora* sp.		39.6	4.7	47.7	6.3	29.9	9.3	6.7	12.1	38.8	1.9
*Scenedesmus quadricauda*		0	0	0	3,6	0	4.1	0	0	0	11.1
**Bacillariophyta**											
*Diatoma ehrenbergii*		1.4	15.1	0.8	9.8	0	12.4	0	6.6	0	3.7
*Navicula* sp.		7.2	18.6	4.7	15.2	3.1	9.3	25.8	15.4	3.7	4.6
*Nitzschia* sp.		12.9	17.4	3.1	8.9	5.5	11.3	7.5	8.8	1.5	13.0

**Table 3:** A list of the parameters measured at the sampling sites at the sampling of phytobenthos and phytoplankton; TI (trophic index) and SI (saprobic index)—both calculated on the base of diatoms; EC—electric conductivity of the water; Abbreviations: W—winter, S—summer; VR—HPP Vrhovo reservoir; BO—HPP Boštanj; BL—HPP Blanca; KK—HPP Krško; BR—HPP Brežice. SI—saprobic index, TI—trophic index. We used blue color for winter samples and yellow for summer to support the differeces with graphic.

	VR_W	BO_W	BL_W	KK_W	BR_W	VR_S	BO_S	BL_S	KK_S	BR_S
Consecutive position of the reservoir downstream	1	2	3	4	5	1	2	3	4	5
T of water [°C]	8.1	7.8	9.0	8.6	10.2	24.2	25.0	22.1	21.0	24.4
pH	8.0	8.0	8.1	8.1	8.1	7.7	7.5	7.8	8.2	8.4
O_2_ [mg/L]	14.0	12.7	13.5	13.8	12.4	8.0	8.1	8.4	8.5	9.7
saturation with O_2_ [%]	116	105	114	117	109	92	93	86	92	112
EC [µS/cm]	430	432	424	417	422	320	311	244	382	378
NO_3_^−^ [mg/L]	7.4	6.6	6.1	5.7	4.6	5.2	5.6	5.8	5.4	5.1
PO_4_^3−^ [mg/L]	0.08	0.08	0.05	0.04	0.03	0.02	0.02	0.02	0.02	0.01
SI value	2.03	1.85	1.94	2.05	1.90	1.86	1.93	1.84	1.89	1.87
TI value	2.98	2.77	2.96	2.88	2.84	2.82	2.90	2.71	2.79	2.72

**Table 4:** The influence of environmental factors on the composition of different communities of primary producers, namely, the phytoplankton and phytobenthic diatom communities, respectively, and phytoplankton and phytobenthic communities of all algae. RDA—redundancy analysis, CCA—canonical correspondence analysis; %TVE—proportion of the total variance explained by the set of statistically significant variables (*p* < 0.05).

Community	Phytoplankton—Diatoms		Phytobenthos—Diatoms		Phytobenthos		Phytoplankton	
Gradient Analysis	RDA		RDA		RDA		CCA	
Parameter	*p* Value	% TVE	*p* Value	% TVE	*p* Value	% TVE	*p* Value	% TVE
Water temperature	0.002	65	0.002	42	0.002	36	0.002	26
pH	0.036	12						
NO_3_^−^			0.004	15				
Order of the reservoir downstream					0.044	12		
PO_4_^3−^							0.044	15

**Table 5:** Distribution of macrophytes at an individual sample site in the reservoirs and the predominant substrate. The abundance of taxa after Kohler and Janauer 1995: 1—very rare, 2—rare. Abbreviations: VR—HPP Vrhovo reservoir; BO—HPP Boštanj; BL—HPP Blanca; KK—HPP Krško; BR—HPP Brežice.

Reservoir	VR	BO	BL	KK	BR
turbidity	moderate	moderate	moderate	moderate	low
Substrate on the bottom	silt and clay, gravel	boulders, cobbles, silt and clay	boulders, rocks, silt and clay,	cobbles, silt and clay	cobbles, silt and clay
Substrate on the bank	boulders, rocks	boulders, rocks, concrete	boulders, rocks	boulders, rocks	boulders, rocks
Nr. of Species	5	4	4	10	7
*Myriophyllum spicatum*	2	2	2	1	1
*Elodea nuttallii*			1	2	2
*Potamogeton crispus*	1				
*Potamogeton nodosus*			1		
*Potamogeton perfoliatus*				1	1
*Potamogeton trichoides*	1				1
*Lemna minuta*				1	1
*Agrostis stolonifera*					1
*Iris pseudacorus*	1			1	
*Leersia oryzoides*				1	
*Phalaris arundinacea*	1	1		1	
*Phragmites australis*				1	
*Polygonum mite*		1		1	1
*Polygonum hydrolapathum*			1		
*Rorippa amphibia*		1		1	

**Table 6:** Coordinates of the sampling sites.

Name of the HHP Reservoir	Abbreviation	N (° Latitude)	E (° Longitude)
HHP Brežice	BR	45.90242	15.58350
HHP Krško	KK	45.97958	15.48027
HHP Blanca	BL	45.99400	15.36078
HHP Boštanj	BO	46.02005	15.27643
HHP Vrhovo	VR	46.04599	15.21470

## Data Availability

Data can be provided upon reasonable request.

## Supplementary Materials

The following supporting information can be downloaded at: https://www.mdpi.com/article/10.3390/plants14111665/s1, Figure S1: Descriptions, aerial photographs and photographs from the sampling sites on the studied reservoirs. Table S1: All algae in phytoplankton as the number of cells found in samples, Table S2: Diatoms in phytoplankton as the number of frustules found in permanent slides, Table S3: All algae in phytobenthos as the number of cells found in samples; Table S4: Diatoms in phytobenthos as the number of frustules found in permanent slides.
