# Supplementary material for: Communities of Primary Producers in the Series of Reservoirs on the Sava River (Slovenia)
Source: Plants (Basel). 2025 May 30;14(11):1665. doi: 10.3390/plants14111665 (PMC12158048; doi:10.3390/plants14111665)
Supplement: Supplementary file 1 [file plants-14-01665-s001.zip › plants-3622765-supplementary.pdf]

Figure S1 - Descriptions, aerial photographs of the reservoirs and photographs from the sampling sites on the studied reservoirs

All five HPPs are of impoundment type with large dams.	Aerial photos of the lower parts of the studied reservoirs including the HPP dams. Red dots represent the positions of the sampling sites (Maps prepared by M. Holcar).	Photography taken at the sampling site from the bank of the specific reservoir.
The reservoir above the HPP Vrhovo is the first in the series of HPPs on the lower Sava river.<br><br>Operates since 2009.	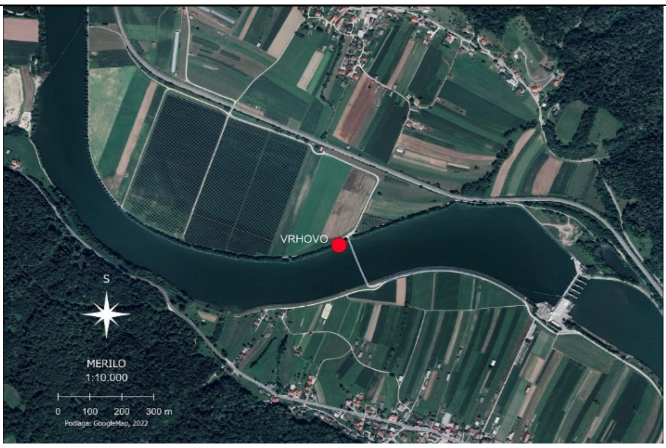	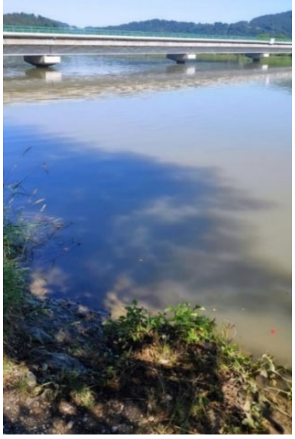<br>HPP Vrhovo reservoir.
The reservoir above the HPP Boštanj is the second in the series.<br><br>Operates since 2006.	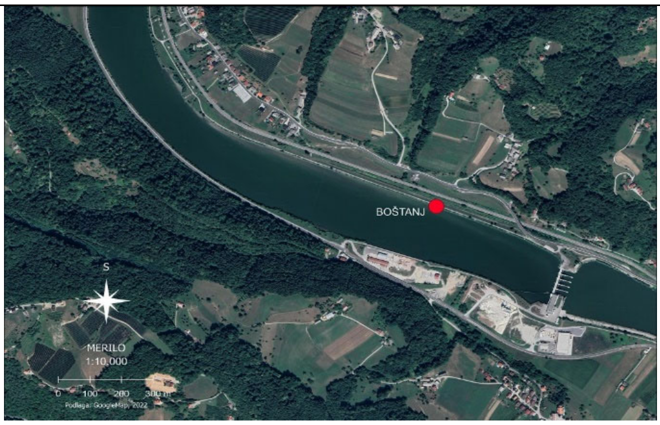	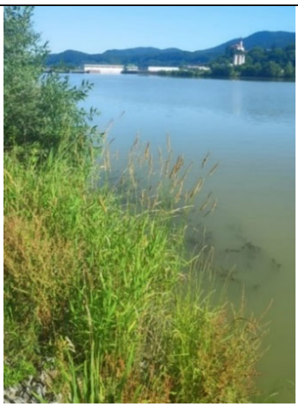<br>HPP Boštanj reservoir.
The reservoir above the HPP Arto-Blanca is the third in the series.<br>Bedrock is made of flysch, layers of limestone and chalk-marl .<br><br>Operates since 2009.	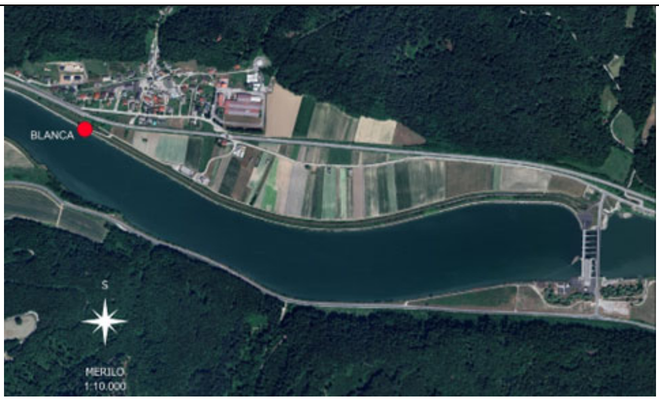	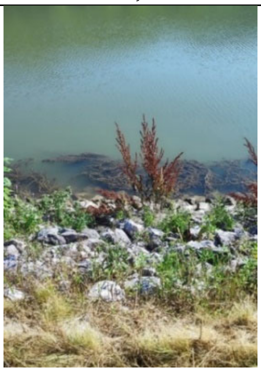<br>HPP Arto-Blanca reservoir.
HPP Krško is the fourth in the series of HPPs.<br>Bedrock is made of dolomite and various Cretaceous sediments.<br><br>Operates since 2013.	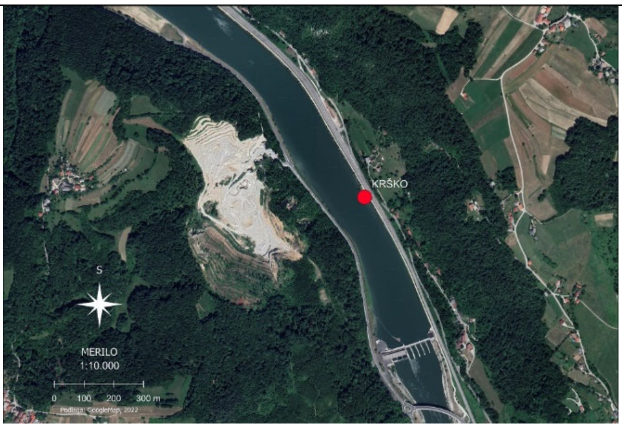	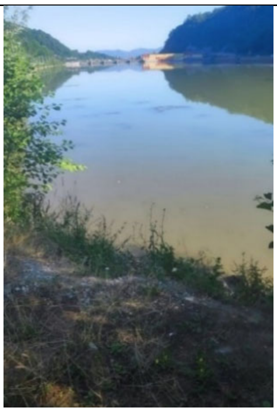<br>HPP Krško reservoir.

The reservoir above the HPP Brežice is the last in the series of HPPs on the lower part of the Sava River.

Operates since 2017.

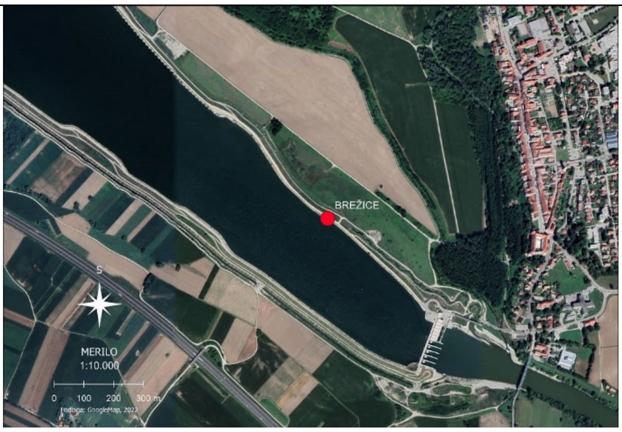

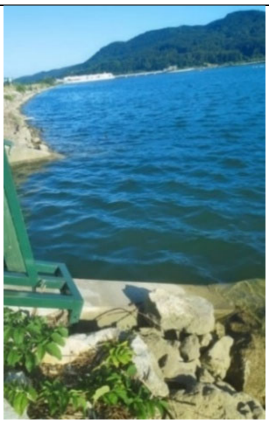

HPP Brežice reservoir.

Table S1 - All algae in phytoplankton as the number of cells found in samples
Abbreviations: VR – HPP Vrhovo reservoir; BO - HPP Boštanj; BL - HPP Blanca; KK - HPP Krško; BR - HPP Brežice reservoir.

Taxa		winter					summer				
		VR	BO	BL	KR	BR	VR	BO	BL	KR	BR
Cyanophyta	*Chroococcus* sp.		2								
	Lyngbya sp.				5						
	Microcystis sp.		1	1			1	1		3	2
	Oscillatoria sp.		1								
	Phormidium sp.	1				14	9	3	3	4	3
	Plectonema sp.	2	1								3
Chlorophyta	*Chlorococcus* sp.										2
	Closterium sp.						1			1	
	Coelastrum sp.	1	2	1	8	10	9	4	36	14	3
	Microspora sp.	15	8	23	7	64	13		15	14	4
	Monoraphidium sp.								2		1
	Pediastrum duplex							19		16	32
	Scenedesmus sp.						4	4	16	16	32
	Scenedesmus quadricauda						12	32	16	12	16
	Spirogyra sp.		2								
	Tetraedron sp.							1			1
	Tetraspora sp.					8					
Bacillariophyta	*Achtantidium* sp.				2	1					
	Amphora sp.		1			1					1
	Cocconeis sp.	1	1	1	1					1	4
	Cyclotella sp.	2		4	3	3	17	16	11	18	25
	Cymatopluera solea		2	2	1				1	6	
	Cymbella sp.			1		3			1	1	
	Diatoma vulgaris	14		4	13	7	10	19	10	8	3
	Diatoma ehrenbergii			2	1		2	6	6	3	1
	Didymosphenia sp.		2								
	Encyonema sp.			1			1	2	3	1	
	Fragillaria sp.	8		4	4	2		2	2	4	
	Fragillaria acus			1		2		1	2		
	Fragillaria ulna				3		2	4	2	1	

	Gomophonema sp.	1			1		1			1	
	Gyrosigma sp.		2	1	2	1				2	
	Navicula sp.	29	7	15	15	11	8	2	1	1	1
	Navicula lanceolata			1	5	3					
	Nitzschia sp.	7	17	16	6	6	4	4	5	4	5
	Nitzschia regula								1		
	Melosira varians	3		2	3	4	2		2	3	12
	Meridion sp.	1									
	Rhoicosphenia sp.	1		1		1					
	Surirella sp.	5	21	1		1	4			2	1
Dinophyta	*Peridinium* sp.	2	1		1		2			2	
Xanthophyta	*Vaucheria* sp.		1								

Table S2 - Diatoms in phytoplankton as the number of frustules found in permanent slides
Abbreviations: VR – HPP Vrhovo reservoir; BO - HPP Boštanj; BL - HPP Blanca; KK - HPP Krško; BR - HPP Brežice reservoir.

	**winter**					**summer**				
Species	VR	BO	BL	KK	BR	VR	BO	BL	KK	BR
Achnanthidium pyrenaicum						3	4	4	5	2
Cocconeis placentula						6	4	5	3	2
Cyclotella meneghiniana		2			1		10	9	11	17
Cymatopleura elliptica									1	
Cymatopleura solea						2				
Cymbella compacta								1		
Cymbella lange-beralotii										2
Diatoma ehrenbergii	1					10	13	10	12	9
Diatoma tenuis						1				
Diatoma vulgaris		1		1		6	6	7	5	1
Encyonema minutum						1	3	2		
Encyonema silesiacum						3		1		
Fragilaria acus							1	1		
Fragilaria ulna						3		1	2	2
Gomphonema olivaceum						2	1		2	1
Gomphonema olivaceolacuum								2		
Gomphonema clavatum										2
Gyrosigma attenuatum								1		
Gyrosigma sciotense									3	1
Melosira varians								1	4	2
Navicula capitatoradiata		1								
Navicula lanceolata			1	1	1	3	1	2	1	2
Navicula tripunctata	2	1					5	1		3
Navicula trivialis						2				
Navicula veneta										1
Nitzschia brevissima						1				
Nitzschia dissipata						2	1			2
Nitzschia palea							1			

Nitzschia recta				1						
Nitzschia regula						2			1	
Nitzschia tabbellaria								1		
Nitzschia tenuis								1		
Surirella angusta						1				1
Surirella brebissoni						2				

Table S3 - All algae in phytobenthos as the number of cells found in samples
Abbreviations: VR – HPP Vrhovo reservoir; BO - HPP Boštanj; BL - HPP Blanca; KK - HPP Krško; BR - HPP Brežice reservoir.

		**winter**					**Summer**				
		VR	BO	BL	KR	BR	VR	BO	BL	KR	BR
Cyanophyta	*Lyngbya* sp.	4	9	5	7	19		5	1	5	6
	Oscillatoria sp.	17	28	43	35	41	18	19	9	12	16
	Phormidium sp.						2		5	3	
	Plectonema sp.			3				3	1		
Chlorophyta	*Chlorococcus* sp.										4
	Coelastrum sp.	3		10						2	2
	Microspora sp.	55	61	38	8	52	4	7	9	11	15
	Monoraphidium sp.							1	1	1	
	Scenedesmus sp.								8	8	4
	Scenedesmus quadricauda							4	4		12
	Spirogyra sp.		2	2		3					
	Tetraedron sp.				1						
	Tetraspora sp.					1					4
Bacillariophyta	*Achtantidium* sp.									1	
	Amphora sp.	2	1		1					1	
	Cocconeis sp.	1	1		1		3	2	1	1	1
	Cyclotella sp.			2	1		1	1		1	
	Cymatopluera eliptica				1						
	Cymatopluera solea	1	1			1					
	Cymbella sp.	1			1			1	1	2	2
	Denticula tenuis				1						1
	Diatoma vulgaris	2	1	1			6	7	6	3	3
	Diatoma ehrenbergii	2	1				13	11	12	6	4
	Encyonema sp.			1	2		3	5	4	1	1
	Fragillaria sp.				3	2			4	2	3
	Fragillaria acus	2	1						1		
	Fragillaria ulna	3			2	1		3	3		5
	Gomophonema sp.	2	2		2			2	1	1	1
	Luticola sp.							2			
	Navicula sp.	10	6	4	31	5	16	17	9	14	5
	Navicula capitatoradiata							4	1	3	1
	Navicula lanceolata	13	2	1	7	2	2	2	1		
	Navicula reicherdiata		1								
	Nitzschia sp.	18	4	7	9	2	15	10	11	8	14

	Nitzschia brevissima	1	2	6	3	4	1				1
	Nitzschia recta		2	2	2						
	Nitzschia regula	1							1	1	
	Nitzschia tabbelaria		1								
	Melosira varians						1	4	2	1	3
	Rhoicosphenia sp.	1	1				1			1	
	Surirella sp.		1		2	1		2	1	1	
Dinophyta	*Peridinium* sp.			2							
Xanthophyta	*Vaucheria* sp.									1	

Table S4 - Diatoms in phytobenthos as the number of frustules found in permanent slides
Abbreviations: VR – HPP Vrhovo reservoir; BO - HPP Boštanj; BL - HPP Blanca; KK - HPP Krško; BR - HPP Brežice reservoir.

	**winter**					**summer**				
Species	VR	BO	BL	KK	BR	VR	BO	BL	KK	BR
Achnanthes coarctata						1				
Achnanthidium minutissimum	8	4	10	5	6	3	4	3	6	2
Achnanthidium pyrenaicum	34	39	32	26	33	21	28	20	20	18
Amphora copulata							1			
Amphora ovalis		14		2			7	1		1
Amphora pediculus	1		11	10	3	7		1	3	
Cocconeis pediculus		6		3	4	1	5	1	4	2
Cocconeis placentula	34	54	40	16	32	46	43	23	42	45
Cyclotella meneghiniana	5	3	3	3	5	11	28	19	48	48
Cyclotella stelligera										1
Cymatopleura elliptica		1						3		2
Cymatopleura solea	9	1		2	1	2	3	8	3	5
Cymbella compacta	2			1		3	1	3		2
Cymbella lange-beralotii			1		1				2	6
Cymbella proxima		1								
Cymbella tumida	1		1			1				
Denticula tenuis				1		4	1		1	
Diatoma ehrenbergii	8	11	7	5	13	66	38	63	41	35
Diatoma mesodon				1		2	1			5
Diatoma moniliformis	2	4		5	11		2	3	3	
Diatoma problematica				8						
Diatoma tenuis			1		1					
Diatoma vulgaris	17	14	11	17	18	14	23	53	18	27
Eunotia pectinalis								1		
Encyonema minutum	6	15	15	5	9	7	17	11	10	20
Encyonema silesiacum	5	11	4	18	13	8	6	5	15	23
Encyonopsis cesatii					1					
Fragilaria acus					1		3			2
Fragilaria construens		1			2		1			1
Fragilaria leptostauron	1				1					
Fragilaria parasitica						1				
Fragilaria radians		2						1		
Fragilaria recapitellata							1			2
Fragilaria ulna	4	1	5	3	10	9	4	12	9	12
Frustulia vulgaris	5	2		3		4	1	4		1

Gomphonema olivaceum	8	12	16	4	11	10	8	6	6	7
Gomphonema clavatum		1	3		2	3	2	2	3	
Gomphonema minutum		3			2	5	4		7	6
Gomphonema occultum		1								
Gomphonema truncatum		1								
Gyrosigma acuminatum	1	2	2	2	3				1	
Gyrosigma attenuatum						3		2		1
Gyrosigma obtusatum	2			1		1	4			1
Hannaea arcus		1						2		
Hippodonta capitata	2					1	1		2	1
Luticola cohnii			23		3	4		1	3	
Luticola goeppertiana	2		4			5	19		3	
Luticola nivalis		3		1	7					
Luticola ventriconfusa		6	10		4		1			
Melosira varians	3	4	1		4		6	15	2	9
Meridion circulare			1		1	2				1
Navicula antonii		2		18		2	2		3	
Navicula capitatoradiata	9	3	3	8	4	16	14	5	8	9
Navicula cryptofallax	1									
Navicula lanceolata	21	22	31	45	29	13	14	20	5	8
Navicula radiosa		5		15	9		1	2	2	2
Navicula reichardtiana	41	24	8	31	8	7	16	15	24	20
Navicula tripunctata	25	9	27	6	21	22	17	22	14	29
Navicula trivialis		3			8					
Navicula veneta	18	9	8	12	5	27	20	9	24	4
Navicymbula pusilla									3	
Nitzschia angustata		7	1				1	3	7	
Nitzschia brevissima		29	71	48	64	3	2	1		4
Nitzschia constricta	3					4				
Nitzschia dissipata	35	23	16	9	5	18	15	14	7	4
Nitzschia palea	29	17	20	19	15	30	20	14	24	11
Nitzschia recta	22	10	5	6	7	6	2	15	7	7
Nitzschia regula	13	2		11	5	3	1	11	4	4
Nitzschia solgensis		3		4					4	
Nitzschia tabbellaria					1			2		
Placoneis placentula				1						
Reimeria sinuata				1			1		1	1
Rhoicosphenia abbreviata	1	4	2		3	2	3	2	2	3
Surirella angusta	3	2		9	3	2	3		5	3
Surirella brebissoni	1	5	6	6	3		3	1		5
Surirella linearis		1	1				1			
Surirella minuta	18	2		9	8		1		4	
Surirella roba								1		
